# Synthetic Biology, Directed Evolution, and the Rational Design of New Cardiovascular Therapeutics: Are We There Yet?

**DOI:** 10.1016/j.jacbts.2023.06.003

**Published:** 2023-07-24

**Authors:** Douglas L. Mann

**Figure:**
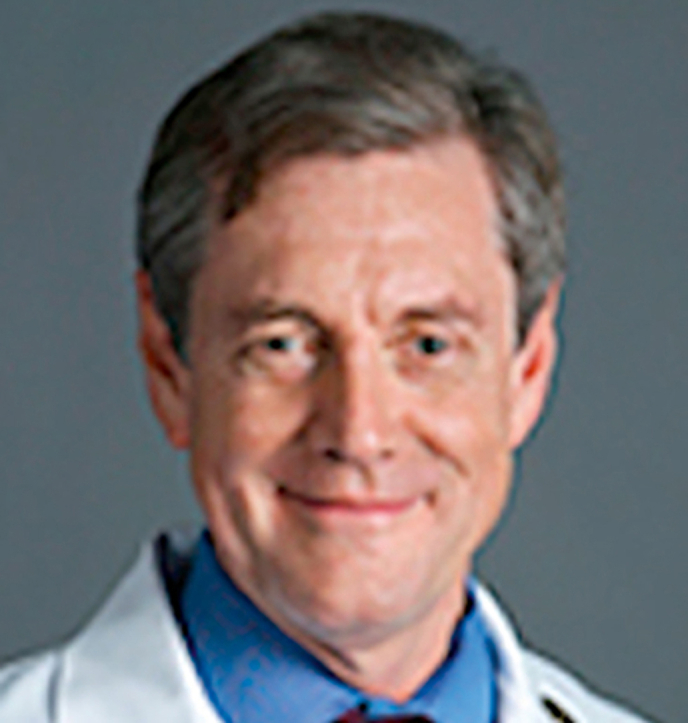

Synthetic biology is a growing field within translational medicine that holds great promise for accelerating the development of new cardiovascular therapies, including (but not limited to) precision medicine, RNA and DNA therapeutics, tissue engineering, and regenerative medicine. Inherently interdisciplinary, synthetic biology combines both biological and engineering principles to create biological systems that are designed to address specific scientific questions. The advent of synthetic biology has changed the way in which scientists think about designing new therapeutic agents by using living cells, as opposed to chemical structures, as the scaffold for designing and developing new medicines.^1^ However, given the inherent complexity of natural biological systems, the application of synthetic biological approaches to the design of new cardiovascular therapeutics has been slow in coming. Recent advances, however, in the field of directed evolution coupled with deep learning may change all this.

In 2018, Frances Hamilton Arnold was awarded one half of the Nobel Prize in Chemistry for her work on engineering proteins and enzymes using the principles of Darwinian evolution to select candidate proteins with desirable properties. Directed evolution refers to an iterative laboratory-based process for creating gene diversification by creating functional gene variants followed by screening and identification of protein variants that have desired properties. As noted by the Swedish Academy of Sciences, “without even knowing they were doing it, humans evolved and optimized enzymes and binding proteins over many generations. Directed evolution of enzymes and binding proteins is a man-made procedure built on molecular insights, which moves the evolution process into the laboratory and speeds it up. The procedure relies on intended variation of protein sequences at a defined level of randomness. This is coupled to engineered screening and selection strategies.”^2^

Typically, the process of defined evolution begins by introducing genetic diversity into a population of organisms (usually bacteria or yeast) that produce the protein of interest. Biological diversity can be created in several ways, but it is most commonly performed through random mutagenesis, which introduces random changes into the gene that encodes the protein. This creates a library of genes that each code for a slightly different version of the protein. The organisms are then allowed to grow and produce their proteins. Once the proteins have been produced, a screening and selection process is used to identify the organisms that produce proteins with the desired traits. The screening process can vary greatly depending on the specific trait being selected for. For example, if the desired trait is an enzyme’s ability to catalyze a particular reaction, the screening process might involve adding a substrate and seeing which organisms are able to convert it into the desired product.

Although the therapeutic applications of directed evolution are still in the early stages, this technique holds promise in several therapeutic areas that are important for patients with cardiovascular disease, such as the following.

• **Enzyme replacement therapies:** Directed evolution can be used to develop enzymes that are more stable or active for use in enzyme replacement therapy for lysosomal storage disease (eg, Fabry disease).
• **Drug development:** Directed evolution can also play a role in drug development. For example, enzymes evolved through this process can be used to synthesize complex drug molecules more efficiently or generate new compounds with novel functions and improved properties.
• **Enhancing tissue repair:** The method of directed evolution has the potential for enhancing immuno-regeneration through the development of chimeric antigen receptor monocyte–derived macrophages that are engineered to phagocytose dying or necrotic myocardial cells, thereby accelerating the resolution of inflammation, and facilitating improved tissue repair.
• **Antibody development:** The concept of directed evolution can also be applied to develop antibody drug conjugates that can bind to specific target cells, such as cardiac fibroblasts, which may allow for enhanced reprogramming of fibroblasts into cardiac myocytes.^3^
• **Gene therapies:** The method could be used in capsid engineering of recombinant adeno-associated virus 9 vectors to improve the targeting of novel gene therapies to cardiac myocytes or other cell types in the heart.

Although directed evolution is becoming an increasingly important tool for the development of new drugs and therapies, there are several important limitations associated with the technique. As one important example, directed evolution relies on iterative cycles of mutation and selection, so the breadth of variants that can be generated in each cycle is relatively small. This can limit the diversity of the molecules produced, potentially excluding some beneficial variants. There is also an inherent selection bias insofar as directed evolution favors the identification of certain types of variants, potentially missing out on other beneficial variants. In addition, the process of directed evolution can be time consuming and resource intensive, and the results are unpredictable. Many proteins are composed of multiple domains that interact with each other. Directed evolution may alter one domain of a protein without considering the allosteric effects on other domains, which could lead to unforeseen changes in the protein‘s function or stability. Finally, there are important issues concerning biosafety. For example, there is a risk that organisms with newly evolved traits could escape from the laboratory and potentially cause harm to natural ecosystems. Given that the origins of SARS-CoV-2 remain uncertain, and that there are ongoing questions about whether that virus might have accidentally escaped from a laboratory in Wuhan, China, the concerns about unforeseen consequences of directed evolution are warranted.

Despite these limitations, directed evolution has found numerous applications in the field of drug development, as well as other aspects of biotechnology. Although there are a number of ongoing technologic advances in the field that are designed to overcome current limitations and make the therapeutic application of developed evolution faster, more predictable, and more effective, at the time of this writing it is too soon to know whether those technologic advances will lead to new cost-effective therapies for treating patients with cardiovascular disease.
